# Extrusion bioprinting: meeting the promise of human tissue biofabrication?

**DOI:** 10.1088/2516-1091/adb254

**Published:** 2025-03-11

**Authors:** Ian Holland

**Affiliations:** 1 Institute for Bioengineering, School of Engineering, The University of Edinburgh, Edinburgh, United Kingdom; 2 Deanery of Biomedical Science, The University of Edinburgh, Edinburgh, United Kingdom; 3 Centre for Engineering Biology, School of Biological Sciences, The University of Edinburgh, Edinburgh, United Kingdom

**Keywords:** extrusion bioprinting, organ printing, biofabrication, technology hype

## Abstract

Extrusion is the most popular bioprinting platform. Predictions of human tissue and whole-organ printing have been made for the technology. However, after decades of development, extruded constructs lack the essential microscale resolution and heterogeneity observed in most human tissues. Extrusion bioprinting has had little clinical impact with the majority of research directed away from the tissues most needed by patients. The distance between promise and reality is a result of technology hype and inherent design flaws that limit the shape, scale and survival of extruded features. By more widely adopting resolution innovations and softening its ambitions the biofabrication field could define a future for extrusion bioprinting that more closely aligns with its capabilities.

## 1. Introduction

**Figure 1.**
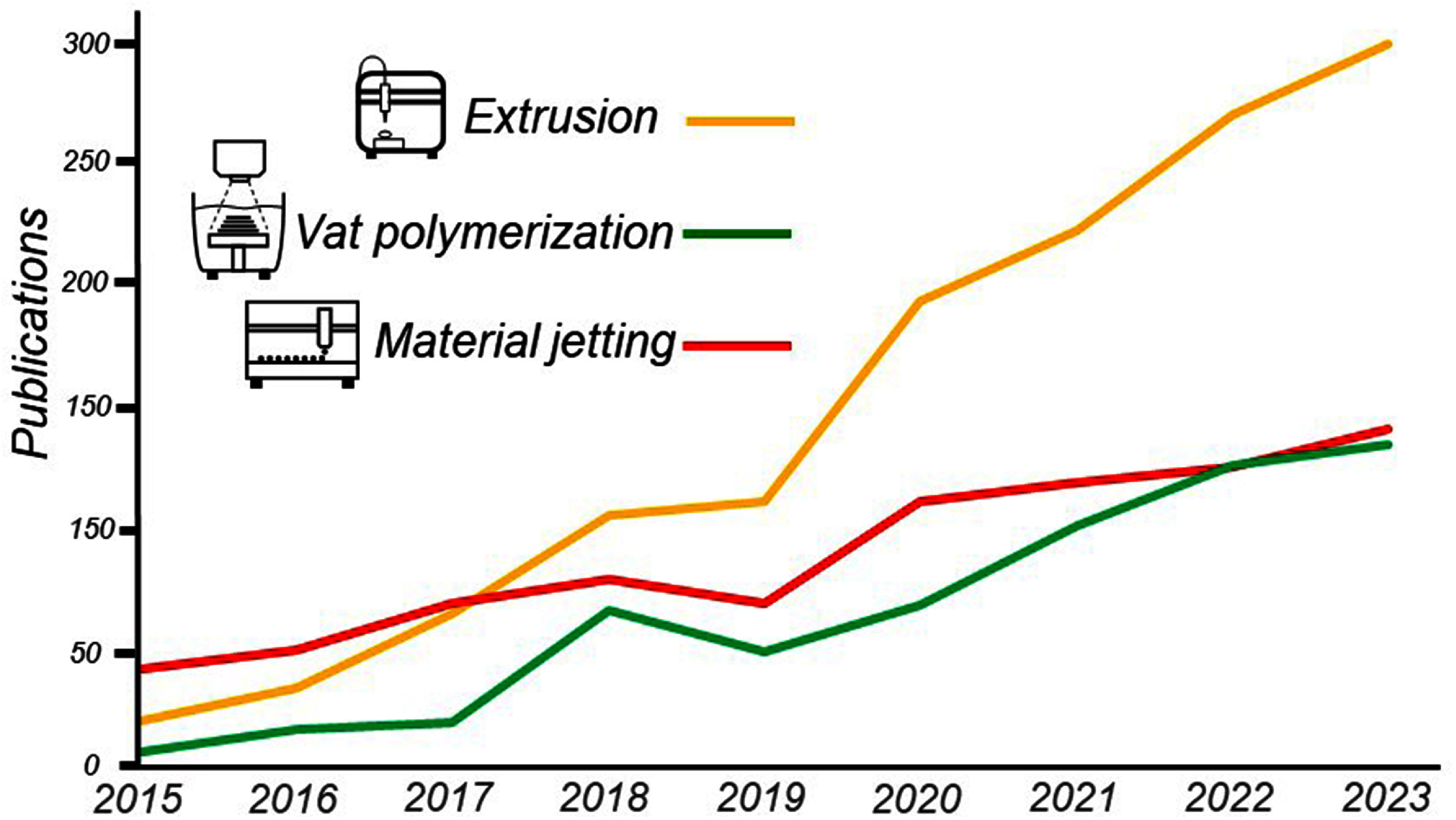
Number of publications for each bioprinting classification between 2015 and 2023. Data taken from a SCOPUS search 9 October 2024 with the term ‘bioprinting’ mentioned in the title or abstract in combination with any of the following keywords for each type. Material jetting—‘inkjet’, ‘ink-jet’, ‘jet’, ‘jetting’, ‘laser’. Vat Polymerization—‘digital light processing’, ‘direct light projection’, ‘light based’, ‘stereolithography’, ‘stereolithographic’, ‘two photon polymerization’, ‘vat polymerization’. Extrusion—‘extruded’, ‘extrusion’.

Biofabrication is a multidisciplinary field that aims to create functional human anatomy for use in research and in the clinic [1]. It has the potential to alleviate many of the economic, ethical and sustainability challenges faced by global healthcare systems [2]. Over the previous decades the biofabrication field has seen the emergence of bioprinters as a platform for the deposition of living cells and matrix mimics into 3D shapes that aim to represent human tissue. There are a range of bioprinting technologies that can be categorised into three groups [3] of material jetting [4], vat-polymerisation [5] and extrusion systems [6]. All of these types, and their variants, present biofabrication researchers with advantages and disadvantages, the discussion of which is the topic of many literature reviews. Inkjet bioprinting, one of the first to be developed, has the advantages of high resolution and low-cost [7, 8] but can experience blockages when using high cell densities [9]. Vat-polymerisation technologies can reach the high resolutions needed for human tissue [5, 10], but currently face challenges to attain heterogeneous structures [11]. As the field of biofabrication has grown, extrusion bioprinting has become the most popular technology and now features in over half of bioprinting publications (figure 1) [12]. It is the rise, promise and capability of extrusion bioprinting that is the focus of this perspective article.

Extrusion bioprinting was first reported in 2002 [13]. It allowed the extrusion of cell-laden hydrogels from a nozzle into a filament positionally deposited into a 3D arrangement defined by a predetermined CAD model [6]. Progressively, the range of cell types and hydrogels that could be extruded, now referred to as bioinks, was expanded [14]. With research growth came increasingly positive references regarding the potential of the technology. In 2013 extrusion bioprinting was termed a ‘disruptive technology’ [15] with industry claims to be on the brink of organ printing [16], predictions of a printed human heart within a decade [17] and that it would alleviate the donor crisis [18]. A large growth in the bioprinting commercial market was also forecasted [19]. These financial projections have been largely realised [20] with an expansion in the number of extrusion bioprinting companies and competition driving down equipment costs. It is common for tissue engineering research groups to have at least one extrusion bioprinter on the laboratory bench. An industrial sector now feeds the resulting demand for hardware, software and consumables [21].

Despite the commercial success, questions remain regarding how close extrusion bioprinted constructs are to human tissue and if the promises will be fulfilled. Extrusion bioprinting is beginning its 3rd decade, attracting a considerable amount of research time and funding throughout this period. It is now within the timeframe for many of the organ printing predictions. In this perspective article the evolution, promises, capabilities and clinical use of extrusion bioprinting are examined. The technology is also placed in the context of the complexity of the human tissue biofabrication challenge and the impact of having such a dominant technology is discussed. It is acknowledged that some of the arguments are controversial and go against the prevailing positive portrayal of extrusion bioprinting in the scientific literature and mass media. However, it is hoped that the presentation of these views will initiate a balanced debate amongst the biofabrication community regarding the future of its leading technology.

## 2. Hype: predictions and perception

Extrusion bioprinting is the most common bioprinting technology in the biofabrication community [12, 14, 22–24]. For some extrusion bioprinting has become synonymous with bioprinting. Media articles [25, 26] and scientific publications [27–44] describe extrusion bioprinting using only the shorthand ‘bioprinting’ term. Reporting on bioprinting has increased greatly and, with extrusion bioprinting being widely acknowledged as the leading technology [12], the majority of bioprinting articles will be referring to extrusion. In the scientific literature and mass media there are many articles highlighting bioprinting advances and making predictions [45–50]. However, it has been recognised that this commentary has frequently moved towards overstatement or hype [51–58]. Positive references towards the potential of bioprinting and the impact it will have on the future of human healthcare are common, especially in regards to organ printing. An analysis of English language bioprinting stories found that 86.7% positively portrayed bioprinting and 32.7% indicated that organ printing would be possible in the near future [59]. Although the mass media articles analysed were not subjected to peer review, such hyperbole is ultimately derived from the scientific literature and also academic press releases, which have been identified as a contributor towards the general trend of scientific hype [60]. There is also evidence of a general increase in positive wording across all disciplines in research papers [61] and grant applications [62]. In the scientific literature, extrusion bioprinting has been promoted, explicitly and implicitly, as the route towards whole-organ printing for transplantation [18, 63–67]. Mass media articles will then repeat and amplify these claims [59]. There are also examples of hype moving towards falsehood with statements, in scientific publications, that bioprinting is currently able to build organs [64, 68–71].

Alongside the positive words are frequently diagrams and animations depicting macroscale human organ printing, including kidneys [72, 73], livers [74], hearts [75], lungs [76] and brains [77]. Concept illustrations are not limited to the mass media but also appear in scientific publications [18, 66, 78, 79]. There are concerns that this imagery is overselling the technology to the public [80]. Images of actual extruded anatomical shapes have also promoted the technology to the wider population. Among the first reported, in scientific and then media publications, were outer ears [81] and noses [82]. Publicity from these pioneering studies has played an important role in elevating the public perception of the technology. The inclusion of organ printing in popular science fiction stories may be also have raised awareness with the notion that the concept of 3D bioprinting could have originated in this genre [83–85].

An indication of the positive viewpoint that the public have of bioprinting has been shown in a survey and focus group study [86]. Participants were presented with the hypothetical option of replacing their own organ with a living donor organ, a deceased donor organ, a mechanical organ, a xenotransplant organ or a bioprinted organ. The most popular option chosen was bioprinting. Whilst there may be ethical, social and cultural influences, it reveals a preference for a technology unproven in humans over those that, to some extent or another, have. Other research has shown that members of the public consider bioprinting to be a current, clinically available technology, with accounts of patients directly contacting researchers to request their own printed tissue [80]. This highlights the cumulative impact that positive visual and written communication has had on the public perception of bioprinting.

The exaggeration process elevating expectations is referred to as a 3D bioprinting ‘hype-cycle’ [87]. This has been identified as a reciprocal relationship between media outlets and laboratories where the desire for attention from both leads to inflated claims about bioprinting. Evidence also exists that active researchers in the field understand that the potential of bioprinting has been overstated. In a study anonymously interviewing bioprinting research scientists, many stated a concern with the widening gap between the presentation of bioprinting in the media and the state of research progress [80]. Privately held hype concerns are rarely expressed in the scientific literature or the mass media where positive reporting dominates the conversation surrounding the technology.

## 3. Extrusion for the clinic: direction and demand

Publications commonly state that a future use of extrusion bioprinting will be human tissue and whole-organ printing for surgical use [88–90]. An examination of the number and type of extrusion bioprinted human tissues in clinical trials can indicate how close to realisation these predications are. All therapeutic interventions must undergo rigorous assessment in clinical trials before use in humans. Success rates depend on the trial type and technology but are generally low, fewer than 14% of drug candidates reach completion [91]. Despite these low probabilities, beginning a clinical trial is often used as a marker of a technology’s progress towards the clinic.

Bioprinting studies have reached the clinical trial stage. At the time of writing there are 11 clinical trials that use bioprinting technology in any context [92], from a total of over 50 000 trials [93]. Of these bioprinting trials, 4 aim to implant tissue with the others building *in-vitro* models. The type of bioprinting technology used in each trial is not always revealed, however one implant trial can be identified as using extrusion bioprinting, for the previously discussed auricular reconstruction [94]. Progression to clinical trials has been possible because the project correctly exploits the capability of extrusion bioprinting to create complex 3D macroscale shapes. Such an approach aligns with the clinical objective, as the role of auricular tissue is dependent on the macroscale shape, providing an aesthetic patient benefit and allowing the determination of sound location [95]. One of the *in-vitro* model trials can be identified as using extrusion bioprinting to print preclinical colorectal cancer constructs [96]. This project has also progressed to the clinical trial phase by aligning the advantages of extrusion bioprinting with the target application. In this case using the automated dosing capacity to increase experimental throughput and reproducibility [96, 97].

These two studies demonstrate that the technology could have an impact beyond the research bench. However, none of the ongoing trials bioprinting trials are creating the tissue needed to realise the promises of organ printing and relieve the donor crisis. Extrusion bioprinting of heterogenous tissue with the essential microscale organisation that forms the majority of human anatomy has not progressed to clinical trials. Considering the age of technology and the organ printing promises made for it the number of extrusion bioprinting clinical trials is low. The current and future clinical use of extrusion bioprinting contrasts with the confidence portrayed in the mass media and scientific literature.

**Figure 2.**
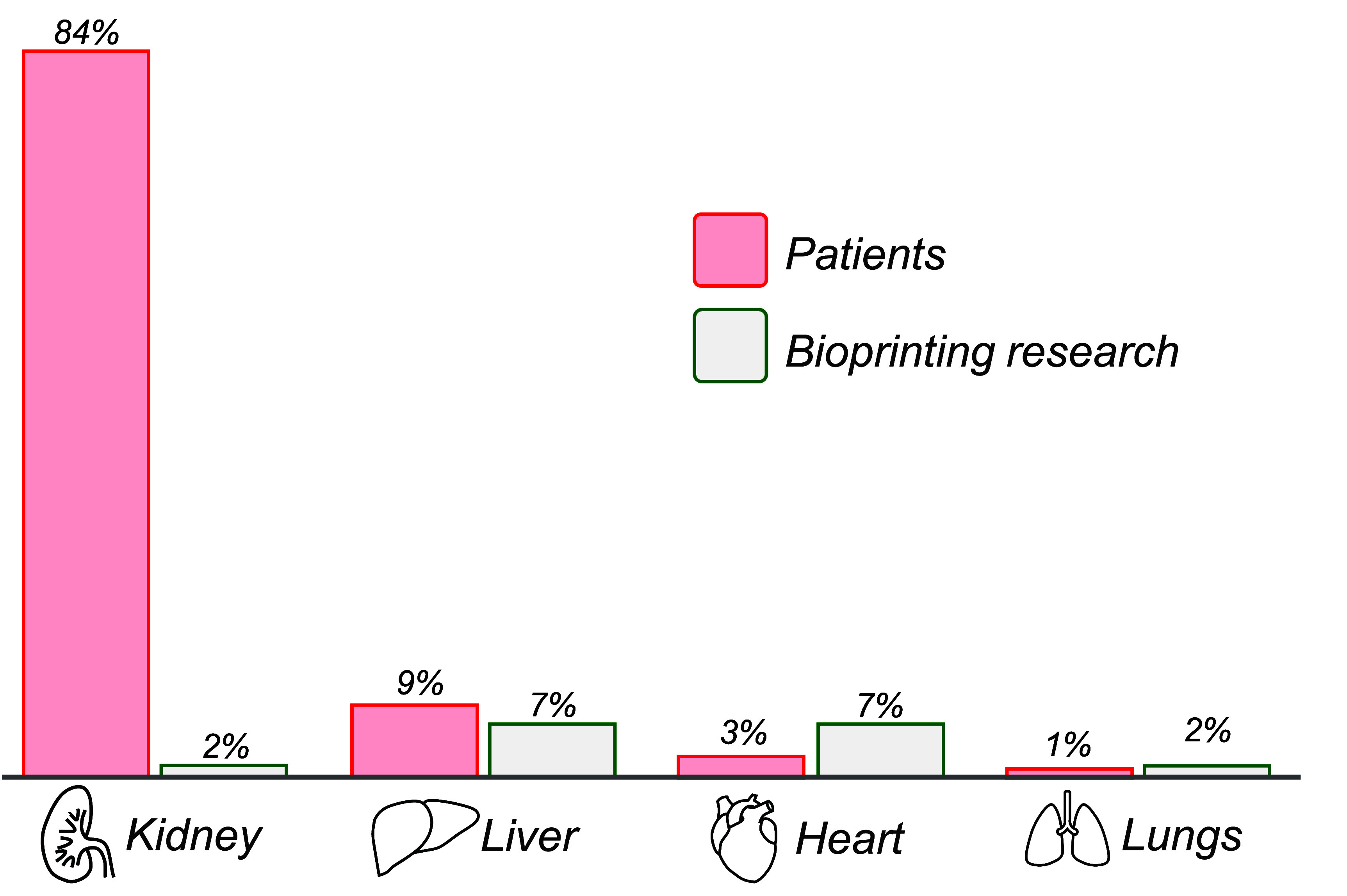
Percentage of patients on transplant waiting list by tissue type (red) and percentage of bioprinting publications featuring those tissues (green). Transplant data shown for September 2023 [98] and bioprinting data for publications up to 2020 [99].

Analysis of the clinical tissue demand can indicate where research is needed and then compared against the direction of existing bioprinting research. The global organ shortage crisis forces patients to be placed on transplant lists while they wait for a suitable donor. Data from the US shows that the most needed organs are kidneys, livers, hearts and lungs, and these types account for 97% of those waiting for surgical intervention [98]. Comparison with data showing the tissue types where bioprinting research is directed reveals that 18% of publications focus on these organs [99] (figure 2) and alternative analysis places this lower at 6.5% [14]. The available data covers the wider field of bioprinting technology, but with extrusion bioprinting being the most common modality it will be very likely to have a similar tissue research allocation profile. The largest discrepancy in the data is for kidneys, needed by 84% of waiting patients but featuring in 2% of bioprinting publications. The complexity of renal tissue is a challenge for extrusion bioprinting with a highly heterogenous microscale architecture [100] making it amongst the most intricate in the human body [101]. The other in-demand tissue types of liver, heart and lungs proportionally receive bioprinting research attention that aligns with their clinical need. However, for field to meet the target of creating organs for transplant, greater percentages above the clinical need will be required.

This data indicates that the clinical demand for organs is not influencing tissue selection for bioprinting research. Studies might be choosing to prove extrusion bioprinting for tissues that are thought as of simpler and focussing on achievable goals during the technology development phase. However, the technology has over 20 years of development history, with its organ printing potential continually advocated. An alternative data interpretation is that 82% of studies avoid the complex tissues most needed by patients, implicitly acknowledging that organ printing may be unachievable. With a research profile directed away from the most needed organs and the simultaneous promotion of organ bioprinting potential, the technology may begin to appear disingenuous. The narrowing of the gap between clinical demand and research effort will require remedial actions from bioprinting stakeholders including researchers, funding bodies and institutions.

## 4. The scale challenge: form and function

The *in-vivo* human tissue that extrusion bioprinting aims to create is highly complex with a hierarchical structure extending from the nano, and microscale levels to larger meso and macroscale organisation [102, 103]. Function follows form and the scale levels combine to imbue tissues with a range of properties and capabilities essential for healthy human life [104]. The smallest nanoscale organisation is inside the cells and the surrounding extra cellular matrix (ECM), where an array of proteins provide functions such as structural support, force transmission and signalling [105]. The multiple cell types are spatially organised within the ECM, combining to make up what have been referred to as microstructural functional units [106]. Organisation within these units is down to the single cell level and can also feature protein aggregates such as structural fibres. Multiple types of these units are then further ordered into larger heterogenous structures to form larger mesoscale units. These finally combine into macroscale tissues that are organised into complete organ systems. It should be acknowledged that this and other descriptions that summarise tissue architecture oversimplify the complexity of biology, particularly the developmental, dynamic and reactionary nature of many tissues.

Vascular tissue, an essential biofabrication target, is a relevant example. At the macroscale vascular vessels have a simple structure of a tube which can vary in dimensions at the millimetre scale. At the next level down the vessel walls are divided into concentric, orientated layers of different cell types, referred to as the tunica adventitia, media and intima. Further complexity exists at a further level down, with subdivisions of these strata into cell-width layers such as the medial lamellar unit and the endothelium [107, 108]. Finally, at the level of the ECM, the proteins that surround the space between the cells are organised into features such as orientated collagen fibres and interconnected elastin layers and struts [109]. Additionally, the ECM composition fluctuates across the width of the vascular wall, with the dominant collagen type [110] and alignment [111] varying according to location. Tissue architecture at this scale is below cell width resolution [112]. As in most other human tissues, all of these hierarchies are essential to the healthy functioning of the vessel, allowing it to not only act as a conduit for blood flow but also to dynamically control blood pressure and having the capacity for self-renewal. The importance of each individual component is highlighted by the impact of genetic diseases that cause the defective assembly of just one element in the hierarchy. Marfan syndrome is one example, which inhibits assembly of the protein fibrillin-1, a sheath for the aforementioned elastin structures, weakening vascular tissue to the point where fatal aneurysms develop [113]. The tissue engineering task is also complicated by considerations such as the high cell densities required to match native tissue [114] and phenotypic cellular variation [115]. Furthermore, the described example of vascular tissue is considered to be one of the easier types to biofabricate with a lower complexity in comparison to other tissues [56]. It is one component of a vast range of organ systems, each with highly complex macro, meso and microarchitecture organisational levels. The biofabrication challenge is to replicate these types of intricate, dynamic constructs. However, the most commonly used technology in the field is based upon a design developed for manufacturing macroscale, inert components.

## 5. Design evolution: inherited issues

**Figure 3.**
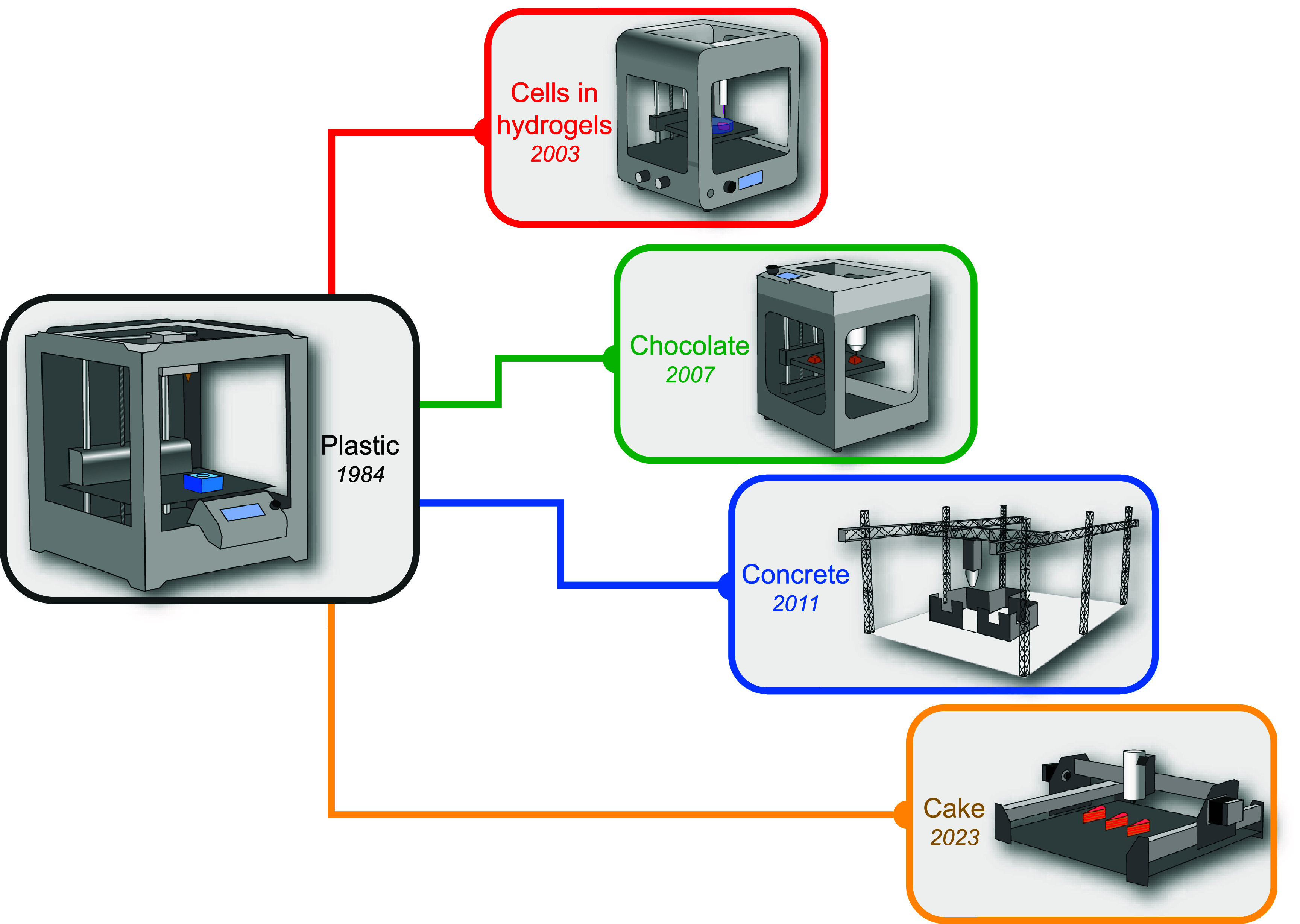
Examples of 3D extrusion printing variants that have evolved from plastic printing and their year of inception. The main differential between the designs is the material extruded. The principle of an extruder mounted onto an x, y, z robot is common throughout.

Extrusion bioprinting has evolved from 3D plastic extrusion printing and this developmental path influences the current biofabrication challenges that the technology faces. Since its invention in 1984 [116] 3D printing has become a popular fabrication tool. A typical system extrudes molten plastic through a nozzle attached to a 3-axis linear drive, positionally depositing a filament in the Cartesian *x, y* and *z* axes [117]. A range of materials beyond plastic can now be extruded including, cake [118], chocolate [119], concrete [120] and cells in hydrogels (figure 3). However, decades after its inception the resolution of 3D printing remains below other fabrication methods, requiring secondary machining technologies for precise features [121, 122]. This is evidenced by the availability of commercial 3D printers with material removal tooling that converts extruded material into high-resolution features [123].

The 3D printing evolutionary design increment that added the ‘bio’ prefix was attained through printhead modifications that extruded liquid phase hydrogels into which cells could be encapsulated. It can be observed that 20 years later there remain few design divergences between ‘bio’ and ‘plastic’ 3D printing. Both technologies have the same basic design principle of a liquid extruding nozzle mounted onto a 3D positional control system. This similarity is exemplified in the commercial bioprintheads that are exchangeable for plastic printheads [124, 125] and the frequent conversion of 3D plastic printers into 3D bioprinters [126–138]. The resolution challenges of extrusion bioprinting, outlined here and by others [139, 140], are a legacy of this historical design increment. A technology developed to fabricate low-resolution homogeneous plastic parts was then applied towards the biofabrication of high-resolution heterogeneous human tissue.

## 6. Resolution: shape, scale and survival

Extrusion bioprinting aims to align the spatial shapes and sizes of extruded cell-laden hydrogels with those observed in native anatomies. The nature of extrusion as a process means that the 3D shape and dimensions of the extruded hydrogel are dictated by the 2D profile of the nozzle orifice. This is commonly a circular shape. Pressure exertion on the bulk liquid in the printhead extrudes a continuous cylindrical hydrogel, referred to as a filament. The external size of the filament is set by the dimensions of the nozzle orifice. Nozzle sizes that match the microscale resolution of the majority of human tissues are not available. The smallest commercial nozzles measure 50 *µ*m (CellInk), with most in the range 100 *µ*m–500 *µ*m [126]. The nozzle forms the filament and the consequentially large filament forms the smallest base structure that the printed tissue can be assembled from [104, 141]. A design feature that has been inherited, as previously discussed. Microscale structures are essential in human tissue and very few are made of cylindrical filament structures that match extrusion bioprinter nozzles dimensions. Skeletal muscle could be considered one exception [38, 142]. The spatial and dimensional mismatch pushes extrusion bioprinting projects to attempt human tissue replication using foundational construction elements that are the incorrect shape and scale.

Direct comparison of the dimensions and shapes of filaments against the microscale structures seen in human tissue can highlight the differences between tissue and technology. As an example the glomerulus, one element of the human kidney, is ellipsoidal in shape and contains a complex network of capillaries with fenestrated endothelia [143]. The outer diameter of the glomerulus is 201 *µ*m [144] and is just one part of the nephron, a further complex arrangement of interconnected ducts and capsules. All these features are below the resolution of extrusion bioprinting and are not composed of a single continuous filament. The numbers needed to reach a functioning organ are also challenging with an adult human kidney containing an average of 617 000 glomeruli [145]. The heterogeneous nature of the kidney is a further challenge, containing up to 73 different cell types [101] and variable ECM compositions [146]. Even extrusion bioprinter designs with multiple bioink cartridges [138, 147, 148] cannot reach this range of cells and materials. From a shape, scale and heterogeneity perspective, extrusion bioprinting is only capable of approximating human tissue complexity.

To reach the resolutions needed the instinctive approach is to reduce the nozzle size, aligning it with the microscale dimensions of human tissue. However, this approach is limited by the problem of survival. Fluid dynamic principles that govern liquid flow through orifices dictate that smaller nozzles require increased extrusion forces, elevating the shear stresses imparted onto suspended cells [149]. Shear stresses decrease cell survival rates [150]. As an example, the viability of extruded endothelial cells suspended in collagen dropped from 86% to 46% on reduction from a 250 *µ*m to a 90 *µ*m nozzle [151]. This example highlights several of the issues that extrusion faces. The smaller nozzle size of 90 *µ*m is nearly an order of magnitude too large to replicate human microtissue architecture and the collagen hydrogel used is classified as a low-viscosity, cell-friendly biomaterial [152]. Additionally, the cited extrusion viability data was published in 2004, indicating the longstanding nature of the problem. A further example of the impact of shear stresses on cellular membrane integrity is laboratory equipment that uses extrusion to break open cells for internal cytoplasm harvesting. Shear stresses can also modify cells that survive the printing process. The sensitivity of cells to shear stresses has caused differentiation [153, 154], reduced proliferation [155], modified gene expression under low forces [156] and inhibited key functionalities such as angiogenesis in endothelial cells [157]. In the context of bioprinting this research demonstrates that extrusion is a damaging process for cells to undergo and that survival should not be the only assessment of their post printed state [6].

Extrusion bioprinting resolution is also influenced by the hydrogel transitioning from the physically constrained environment of the nozzle to the external unconstrained environment. For the primary layer it will be the printed and for subsequent layers the previously extruded substrate. To transition the hydrogel must have rheological properties that permit it flow and exit the nozzle. To assume the intended stiffness of the target tissue and form a firm substrate for subsequent print layers it then needs to change properties and gel. There are a range of gelation mechanisms and these require time. During the gelation phase the hydrogel can continue to flow and divert from the original spatial form [158], often a cylindrical filament. This dynamic process is referred to as filament spreading [159], resulting in a final gelled form that is wider than the nozzle. The final form the hydrogel assumes is also governed by the shape of the substrate it is positioned onto. Filament dimensions are further influenced by the relationships between hydrogel extrusion velocity, the nozzle print speed and the nozzle to substrate distance [160, 161]. Machine learning and in silico modelling approaches have recently helped to understand this challenge, predicting the impact of bioink properties and nozzle geometries on print dimensions and cell viabilities [161–163].

The property of shape fidelity has been proposed as a way to measure the output of the complex interplay between the parameters that contribute to filament spreading. Shape fidelity is defined as the closeness of the extruded structure to the macroscale digital model [6, 164]. However, the aim of biofabrication is to mimic human tissue and the ultimate analysis of printed constructs should be their closeness to the target tissue, or tissue fidelity. Many studies in the extrusion bioprinting field do not assess or discuss how accurately the printed tissue replicates native human tissue. A systematic review of the extrusion bioprinting literature found that over half of studies did not assess the basic attribute of cell viability [165]. An absence of comparison to native tissues can hide low tissue fidelity and also conceal the limitations of the biofabrication technology being used. A combination of technological design flaws and minimal tissue characterisation prevent extrusion bioprinting from reaching the resolutions needed to biofabricate human tissue.

## 7. The 4th dimension: post-printing

Reorganisation post-printing has been proposed as method to transform constructs into structures that better represent tissue [166]. For extrusion bioprinting it would begin with stacked extruded large-scale filaments and leverage the remodelling capabilities of the biomaterials and cells to move towards the *in-vivo* structure. This could be achieved by applying external stimuli, sometimes referred to as 4D bioprinting [167] or be dynamic post-printing processes that are inherent or specifically engineered into the cells and biomaterials. The application of electrical current [168], magnetic fields [169] and temperature [170] are examples of post printing external stimuli. The cellular compaction of collagen [171] and ECM synthesis [172] are examples of dynamic remodelling processes that occur without stimuli. Inherent and stimuli induced processes often mimic those observed in developmental biology, such as folding [173] and growth factor release [174], although they may achieve them via different mechanisms. It is an open question if post-printing rearrangement can transform low resolution, homogenous extruded material into high resolution, heterogenous human tissue. 4D bioprinting has shown a path towards creating shapes that are not possible using extrusion alone [175], but challenges remain. Stimuli responsive biomaterials are mostly only able to perform rudimentary shape changes, respond to one stimuli type [176] and show low cytocompatibility and printability [167]. However, the field is relatively recent and its progress will be applicable across tissue engineering [177] and not limited to extrusion bioprinting. Integrating developmental biology processes and agential materials [178] has the potential to increase the tissue fidelity of extrusion bioprinting. It has been proposed that this strategy will be needed for all bioprinting technologies to reach the aim of functional adult human tissues and organs [179].

## 8. Bioinks: the printability paradox

Rigid extrusion bioprinter hardware configurations mean that the primary method of adapting and aligning to the demands of tissue engineering is to modify the extrudable liquid. Referred to as bioinks, these materials have become a subfield of biofabrication [180]. An aim for extrusion printing bioink studies is to develop ‘printable’ formulations of hydrogels and cells [150, 158, 161, 181]. The definition of printability is itself fluid with no consensus in the biofabrication literature [158, 159]. The challenges are to optimise the rheological properties of a bioink to allow forced extrusion through the nozzle, prevent filament spreading before gelation and maintain low shear stresses for cell survival. These constraints create the ‘biofabrication window’ [6, 182, 183] or printability ‘paradox’ [184]. Printability windows also impact other modalities such as polymer [185, 186] and concrete extrusion [187], but without the difficulty of including living cells. As previously discussed, high-viscosity materials require high nozzle extrusion pressures that elevate shear stresses lowering cell viabilities. Low-viscosity materials spread too readily, causing poor shape fidelity. These constraints, and the resulting experimental space that bioink studies can explore, are imposed by the design features of extrusion bioprinters. Research projects are therefore limited to narrow developmental loops. The focus becomes bioink modification for extrusion bioprinters, rather than developing technologies and materials to replicate human tissue.

**Figure 4.**
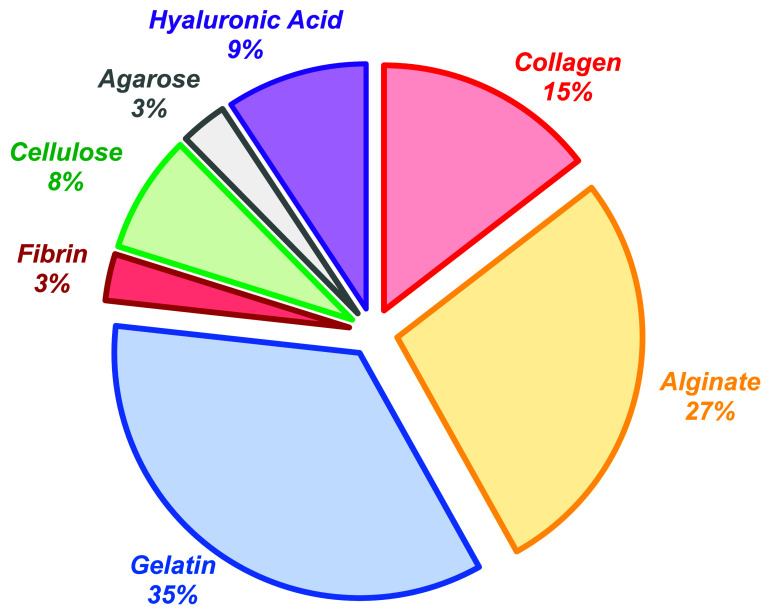
Use of materials in extrusion bioprinting. A SCOPUS search with the terms ‘bioprinting’ and ‘extrusion’ mentioned in the title or abstract text was carried out on 10 October 2024, for articles published in all years. 1475 were returned. Within these primary search results, mentions in the title or abstract text of the following materials was assessed: ‘Alginate’, ‘Cellulose’, ‘Collagen’, ‘Fibrin’, ‘Gelatin’, and ‘Hyaluronic acid’, 1334 articles (90.4%) quoted at least one of these materials. This process created an extrusion bioprinting dataset based on the bioprinting material analysis published in 2022 [194].

Evidence of bioink alignment with the technology ahead of tissue consideration can be seen in the materials selected for extrusion bioprinting studies. Collagen can be used as a bioink material, but low viscosities and slow gelation times make it difficult to print using extrusion bioprinting [188]. However, it is the most abundant human ECM protein [189]. For example, collagen types make up 59% of articular cartilage, 71% of skeletal muscle and 81% of tendon protein composition [190]. To create human tissue, collagen should be prominent in extrusion bioprinting studies. However, data shows collagen usage in 15% of articles (figure 4). Bioinks that are printable but have low human ECM representation are being selected for extrusion bioprinting. Where collagen is used, its chemistry and properties are often altered to enable printing [152] moving it away from the human ECM form. Examples are blending with non-human ECM materials, such as alginate [191] and chemical modification to allow UV light crosslinking [192] with photoinitiators that can be cytotoxic [193]. Printability decisions appear to be aligning bioink compositions with the technology rather than the tissue.

The extrusion bioprinting printability window therefore limits bioink alignment with human tissue. The need to operating inside the printability window has led to the often cited comment that there are not enough bioinks available for the extrusion bioprinting needs of researchers [184, 195–197]. Despite the accuracy of this sentiment, it is caused by the limitations of extrusion and not the concept of using bioinks in tissue engineering. It may be more appropriate to state that there are not enough technologies available for bioinks.

## 9. Design process: inertia and inversion

Extrusion bioprinting has an established presence in many academic and commercial laboratories. Contributing to its popularity is the close relationship between the academic and commercial sides of the technology. Universities and bioprinting companies are often interlinked. Over two-thirds of bioprinting patents are affiliated to a research institution [99] with many university spinout companies featuring academics in board positions and holding investments. An advantage of these relationships is the connection of academia with the research and educational needs of industry [198]. However, the conflicts of interest they generate have created problems in other fields, where the conscious and unconscious favouring of technologies is based on commercial merit [199]. In situations where institutions have financial and temporal investments in a technology the research agenda can become biased [200]. Extrusion bioprinting research could be experiencing the same influences, increasing its popularity. In a scenario where a research group owns an extrusion bioprinter or associated intellectual property, projects can become framed around their use. The technology’s popularity has also been elevated by falling equipment costs [99, 201] and the recent trend to develop low-cost, DIY extrusion bioprinters [135, 202–205]. In combination these factors have allowed extrusion bioprinters to build-up a large amount of academic and commercial inertia. Profit margins and careers have become linked to extrusion bioprinter usage in research and the resulting sales of equipment and consumables.

Extrusion bioprinting popularity may also be responsible for inverting the optimal manufacturing design process. The idealised design manufacturing process begins with the product and then works backwards [206]. The first step aims to obtain a full understanding of the form and function of the product. The process of ideating and designing the fabrication system can then commence, ending with the technology design. However, where a technology already exists there can be pressure to align a current system to suit the product, approaching the manufacturing challenge from the opposite direction. With extrusion bioprinting this scenario occurs frequently with examples where equipment is chosen first and then bioinks modified [207–214]. The cited examples, whilst scientifically rigorous, are part of a trend for technology selection before tissue consideration. Academic publishing pressure may be pushing projects towards this route. Novel technology development requires time that is not always available from typical research grant timelines. With an extrusion bioprinter already in the laboratory, technology development and the route to publication are shortened.

**Figure 5.**
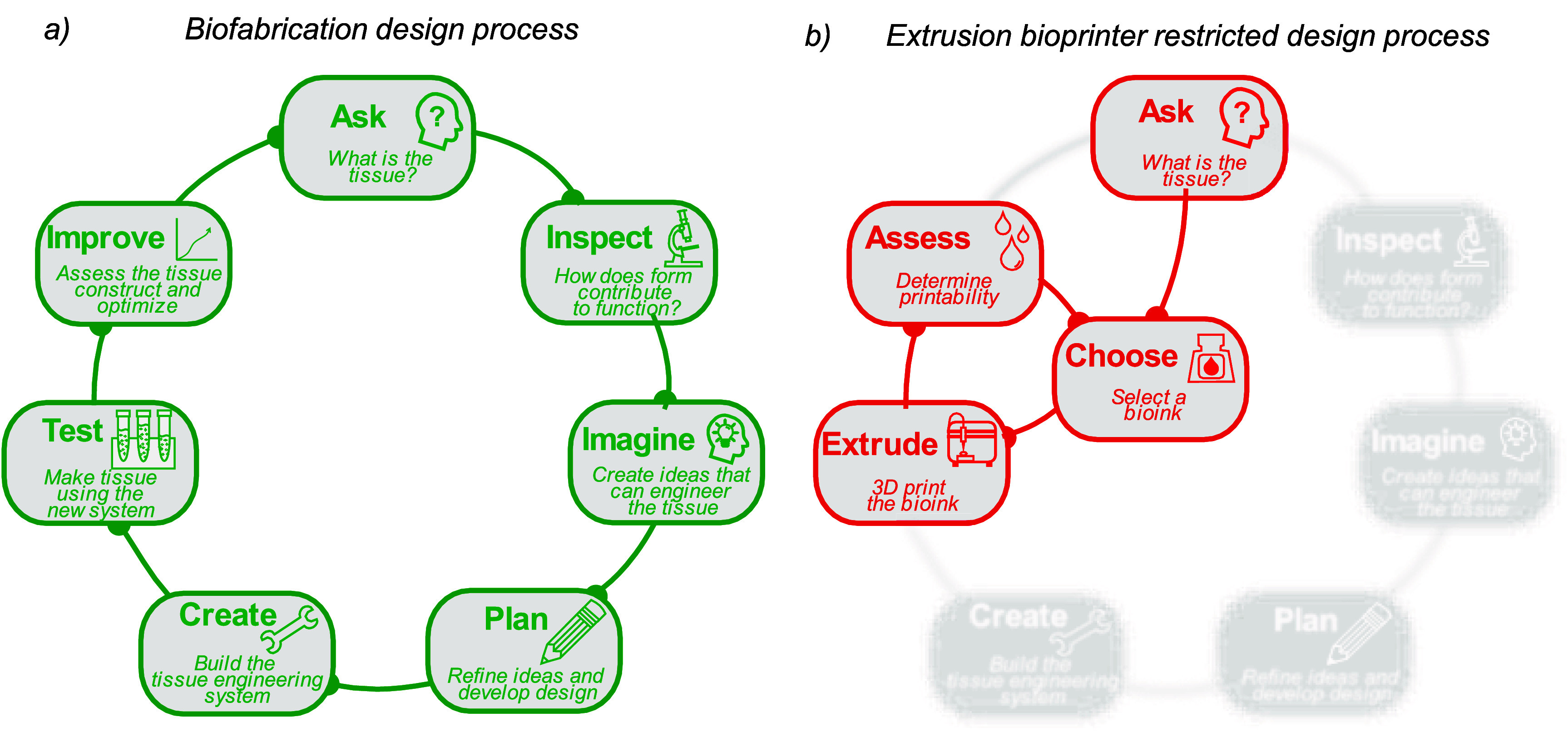
(a) Proposed biofabrication design process and (b) design process limited by early selection of an extrusion bioprinter and bioink combination.

A suggested design process for biofabrication is shown in figure 5(a). First the target tissue is appraised, analysing the micro, meso and macroscale architecture to understand how form contributes to functionality [104]. Extensive anatomical research and advances in imaging technologies have ensured that this information is likely to be published [104, 215]. Next potential biofabrication technologies are assessed for their suitability to build the target tissue. Where they are unable meet the tissue specification requirements, for example in terms of resolution or heterogeneity, then it may be necessary to design, build and test new technologies or combine multiple technologies together. The biofabricated tissue can then be compared to the target tissue and refinements made, repeating the design loop. Biofabrication projects can, due to technological and commercial inertia, choose an extrusion bioprinter-bioink combination from the beginning (figure 5(b)). Early technology path selection limits the innovation available to adapt for replication of the specific tissue. If preselecting an extrusion bioprinter, the primary avenue for adaptation to the tissue is bioink modification. As previously detailed, the bioink developmental window is constrained by the technology, trapping studies in a narrow developmental loop. Similar inhibitory effects will also occur where other biofabrication technologies are selected before the consideration of the target tissue. For extrusion bioprinting, its accessibility allows many groups to contribute to biofabrication research. However, the limitations of the technology constrain the tissue fabrication possibilities of the large research output.

## 10. Suspension extrusion: bioprinting in the bath

An extrusion bioprinting development that may lead to more biomedical applications is suspension bath bioprinting, also known as freeform reversible embedding of suspended hydrogels (FRESH) [216]. The innovation recognises that extrusion is not constrained to the medium of air but can instead be into a liquid support bath. The bath can be a granular [217] or colloidal slurry [218] that is removed following printing or remains as a media to maintain the construct [219]. The innovation significantly improves shape fidelity by reducing the effect of gravity on the filament during gelation [4, 220, 221]. The key benefits are an improvement in resolution and a widening of the bioink experimental space. Features of 20 *µ*m have been printed [222], closer to the microscale dimensions of human tissue needed for biomedical applications. A further suspension bath bioprinting development is collagen printing [223], which as previously discussed is underused in extrusion bioprinting research. Although the technology has challenges, such as forming highly heterogenous tissue, it remains a significant improvement on direct air extrusion bioprinting. To maintain its dominance and find a clinically relevant role extrusion bioprinting will need to more widely adopt suspension bath principles. However, first published in 2015 [216] and cited as the next era in bioprinting [219], the majority of bioprinting publications persist with extrusion in air. Potential reasons could be the additional process steps, materials and expertise that suspension bath bioprinting needs.

## 11. Biomedical applications: alignment with architecture

The biomedical applications of extrusion bioprinting show alignment with the capabilities of the technology. For clinical transplantation it is where macroscale organisation is more important to tissue function than the microscale. Examples are the outer ear, nose [82, 224] and potentially skeletal muscle where the fibrous tissue architecture aligns with extruded filaments [38, 142]. There will, however, need to be some level of high-resolution nervous system integration and vascularisation. The technology is unable to biofabricate tissue types where microscale architecture is essential and these are typically where the greatest disease research and clinical intervention is needed. It is also these tissues that attract the most research funding [225].

A further biomedical role for extrusion bioprinters is cell-hydrogel aggregate printing for *in-vitro* testing, such as preclinical models and drug screening purposes [226], evidenced an ongoing clinical trial [96]. Extruded cell-laden hydrogels are a more representative model when compared to culture on 2D surfaces [227–229]. In this context, the automated capability of extrusion bioprinters is an advantage as macroscale, hydrogel-cell drops can be rapidly deposited into well plates for high-throughput processing [96, 201, 230, 231]. Finally, the use of the technology as a hand-held tool for *in-situ* surgery has recently been explored [232]. Extrusion in this context could have an advantage over existing surgical methods for controlling the application of multiple drug containing hydrogels [147].

## 12. Future prospects: precision and promises

Extrusion bioprinting is beginning its 3rd decade and is no-longer a new technology. With advancing age is a need to deliver on its promises. An understanding that direct air extrusion bioprinting is unable to attain the resolutions needed to create most human tissue has begun to develop [105, 195, 219]. A viewpoint that contrasts with the presentation of the technology in the media and scientific literature. Consequently, the promise and the technological reality of extrusion bioprinting are far apart. The future prospects for the technology will be defined by how this gap will be narrowed. Moderating expectations is a low priority for the biofabrication field, it remains common for papers to promote the organ printing potential of the technology. Articles that highlight hype concerns receive limited attention [59, 80], even when published by field leaders [56].

The key future challenges for extrusion bioprinting are therefore twofold; the promises are too high and the resolutions are too low. To reduce the hype and manage expectations there is a need from academia, industry and publishers to present more realistic ambitions for the technology. Organ printing as a justification for extrusion bioprinting research urgently needs to cease. Emphasis should instead be placed on the technology’s advantages as a testing tool and its research potential to contribute towards the replacement of animal models.

## 13. Conclusion

The theme of this article is understood to be provocative. Can extrusion bioprinting meet its promises of human tissue biofabrication? The answer depends on the promised tissue type and the intended application. Forcing hydrogels through an orifice imposes spatial, rheological, material and cell survival limitations on extrusion bioprinting. The product of this process is a construct of oversized stacked filaments that can only approximate the microscale architecture observed in most human tissue types. These technological hardware flaws are inherited from 3D plastic printing, a technology with similar resolution limitations. The arrival of suspension bath bioprinting provides a direction for increasing extrusion resolutions but remains an underused technology. Emerging results from clinical trials indicate that extruded tissue products that do not require microscale heterogenous organisation can be applied towards a clinical objective. For these examples the promise is aligned to the capabilities of the technology. However, these applications represent a minority of the clinical tissue need and also a minority of the promises made for the technology.

For the majority of the tissue types needed in the clinic, on its own, extrusion bioprinting is not a suitable biofabrication technology. The large organ types required by patients contain microscale heterogenic features that are outside the biofabrication capacity of extrusion bioprinting. To replicate the wide range of intricate human tissue the field is likely to require a correspondingly large range of high-resolution biofabrication technologies. The human tissue challenge is not beyond the capabilities and expertise of the biofabrication field. It is however, largely beyond the capabilities of extrusion bioprinting and a greater acknowledgement, discussion and debate of this situation can only be of long-term benefit to researchers, clinicians and patients.

## Data Availability

The data that support the findings of this study are openly available at the following URL/DOI: https://doi.org/10.7488/ds/7869.
